# Interaction between Hormonal Receptor Status, Age and Survival in Patients with BRCA1/2 Germline Mutations: A Systematic Review and Meta-Regression

**DOI:** 10.1371/journal.pone.0154789

**Published:** 2016-05-05

**Authors:** Arnoud J. Templeton, Laura Diez Gonzalez, Francisco E. Vera-Badillo, Ariadna Tibau, Robyn Goldstein, Boštjan Šeruga, Amirrtha Srikanthan, Atanasio Pandiella, Eitan Amir, Alberto Ocana

**Affiliations:** 1 Department of Medical Oncology, Kantonsspital St. Gallen, St. Gallen, and Faculty of Medicine, University Basel, Switzerland; 2 Translational Research Unit, Albacete University Hospital, Albacete, Spain; 3 Department of Oncology, Queen's University, Kingston, ON, Canada; 4 Department of Medical Oncology, Hospital de la Santa Creu i Sant Pau and Universitat Autònoma de Barcelona, Barcelona, Spain; 5 Divisions of Medical Oncology and Hematology, Princess Margaret Cancer Centre, Department of Medicine, University of Toronto, Toronto, Canada; 6 Department of Medical Oncology, Institute of Oncology, Ljubljana, Slovenia; 7 Centro de Investigación del Cáncer CIC-CSIC, Universidad de Salamanca, Salamanca, Spain; Odense University Hospital, DENMARK

## Abstract

**Background:**

Germline mutations in the *BRCA1* and *BRCA2* genes are the most frequent known hereditary causes of familial breast cancer. Little is known about the interaction of age at diagnosis, estrogen receptor (ER) and progesterone receptor (PgR) expression and outcomes in patients with *BRCA1* or *BRCA2* mutations.

**Methods:**

A PubMed search identified publications exploring the association between *BRCA* mutations and clinical outcome. Hazard ratios (HR) for overall survival were extracted from multivariable analyses. Hazard ratios were weighted and pooled using generic inverse-variance and random-effect modeling. Meta-regression weighted by total study sample size was conducted to explore the influence of age, ER and PgR expression on the association between *BRCA* mutations and overall survival.

**Results:**

A total of 16 studies comprising 10,180 patients were included in the analyses. BRCA mutations were not associated with worse overall survival (HR 1.06, 95% CI 0.84–1.34, p = 0.61). A similar finding was observed when evaluating the influence of *BRCA1* and *BRCA2* mutations on overall survival independently (*BRCA1*: HR 1.20, 95% CI 0.89–1.61, p = 0.24; *BRCA2*: HR 1.01, 95% CI 0.80–1.27, p = 0.95). Meta-regression identified an inverse association between ER expression and overall survival (β = -0.75, p = 0.02) in *BRCA1* mutation carriers but no association with age or PgR expression (β = -0.45, p = 0.23 and β = 0.02, p = 0.97, respectively). No association was found for *BRCA2* mutation status and age, ER, or PgR expression.

**Conclusion:**

ER-expression appears to be an effect modifier in patients with *BRCA1* mutations, but not among those with *BRCA2* mutations.

## Introduction

Mutations in *BRCA1* and *BRCA2* genes explain approximately 15% of familial breast cancers and are the most common hereditary lesions in breast cancer. Compared with women without mutations, those with somatic mutations in *BRCA1* and *BRCA2* genes are at increased risk for the development of breast cancer (life time breast cancer risk of 50–80%). These mutations are inherited in an autosomal dominant fashion and lead to the synthesis of an inefficient protein that impairs DNA repair mechanisms, specifically the homologous recombination pathway [1, 2].

The association between *BRCA* genes and cancer predisposition is well described. There are substantial data showing that after adjustment for stage, breast cancers associated with BRCA mutations are associated with similar outcomes to sporadic breast cancers [3]. Less is known regarding whether survival outcomes are influenced by age at diagnosis of breast cancer, or degree of estrogen receptor (ER) or progesterone receptor (PgR) expression. Here we aimed to identify studies evaluating mutations in *BRCA1* or *BRCA2* genes and clinical outcome in breast cancer to assess the influence of age and hormonal receptor expression on survival. We hypothesized that *BRCA* mutation carriers with ER-negative tumors have better outcomes than women with *BRCA* wildtype and ER-negative tumors.

## Methods

This analysis was conducted in line with the Preferred Reporting Items for Systematic Reviews and Meta-Analyses (PRISMA) guidelines (S1 Table) [4].

### Data sources and searches

Medline (Host: PubMed) was searched for studies published between January 1998 and February 2016), which evaluated DNA repair pathways in breast cancer. We used the MeSH terms ((((BRCA1) AND BRCA2) AND Breast cancer) AND survival) and added the limitation of human studies. The search was restricted to publications in English. Additional studies were identified through reviews of citation lists.

### Study selection and data extraction

Two reviewers (LD, AO) independently evaluated all the titles identified by the search strategy. The results were then pooled and all potentially relevant publications retrieved in full and assessed for eligibility. Disagreement was resolved by consensus. For the primary analysis the following inclusion criteria for selection of studies were used: (i) studies in breast cancer reporting outcomes for overall survival (OS) in patients with *BRCA1* or *BRCA2* germline mutations compared to no such mutations (ii) availability of an adjusted hazard ratio (HR) for the mutational status with its 95% confidence interval (CI) or the associated *p*-value.

The following information was captured using predesigned data abstraction forms: First author, year of publication, BRCA type (if available), age of patients at diagnosis, duration of follow-up, number of patients with and without *BRCA* mutation, proportion of tumors with ER and PgR expression, proportion of patients with metastatic disease. Furthermore, HRs for mutational status from multivariable models with 95% CI and/or *p*-value and the variables used for adjustments were captured.

### Data synthesis and statistical analyses

The primary outcome of interest was OS. The overall effect of BRCA mutational status on overall survival was assessed in a meta-analysis. Estimates of HRs were weighted and pooled using the generic inverse variance and random-effect model. All meta-analyses were conducted using RevMan 5.2 analysis software (Cochrane Collaboration, Copenhagen, Denmark). Statistical heterogeneity was assessed using Cochran’s Q and I^2^statistics and considered present if the *P*-value for Cochran’s Q was <0.10 or I^2^> 50%. Initial analysis was conducted for all studies with available adjusted HRs of overall survival for germline *BRCA* mutational status (*BRCA1* and *BRCA2* combined). Subsequent analyses explored the individual effect of *BRCA1* or *BRCA2* or unspecified *BRCA*. Differences between subgroups were assessed using methods described by Deeks et al. [5]. Sensitivity analyses were done with exclusion of studies that reported HRs which were not adjusted for age or hormonal receptor status. Meta-regression was conducted to explore the influence of age and ER/PgR expression on the association between *BRCA* mutation status and survival. Specifically, a linear regression weighted by total study sample size (weighted least square regression) was carried out to evaluate the impact of median/mean age at diagnosis or proportion of patients who were ER/PgR-positive on the HR for survival for *BRCA* mutational status. Once again, the primary analysis of a pooled HR for *BRCA1* and *BRCA2* was used, if both were reported in the same study. Subsequent analyses were done separately for *BRCA1* and *BRCA2* mutations. Meta-regression analyses were done using SPSS version 20 (IBM Corp, Armonk, NY). All statistical tests were two-sided, and statistical significance was defined as p<0.05. No corrections were made for multiple testing.

## Results

### Characteristics of studies

**Fig 1:**
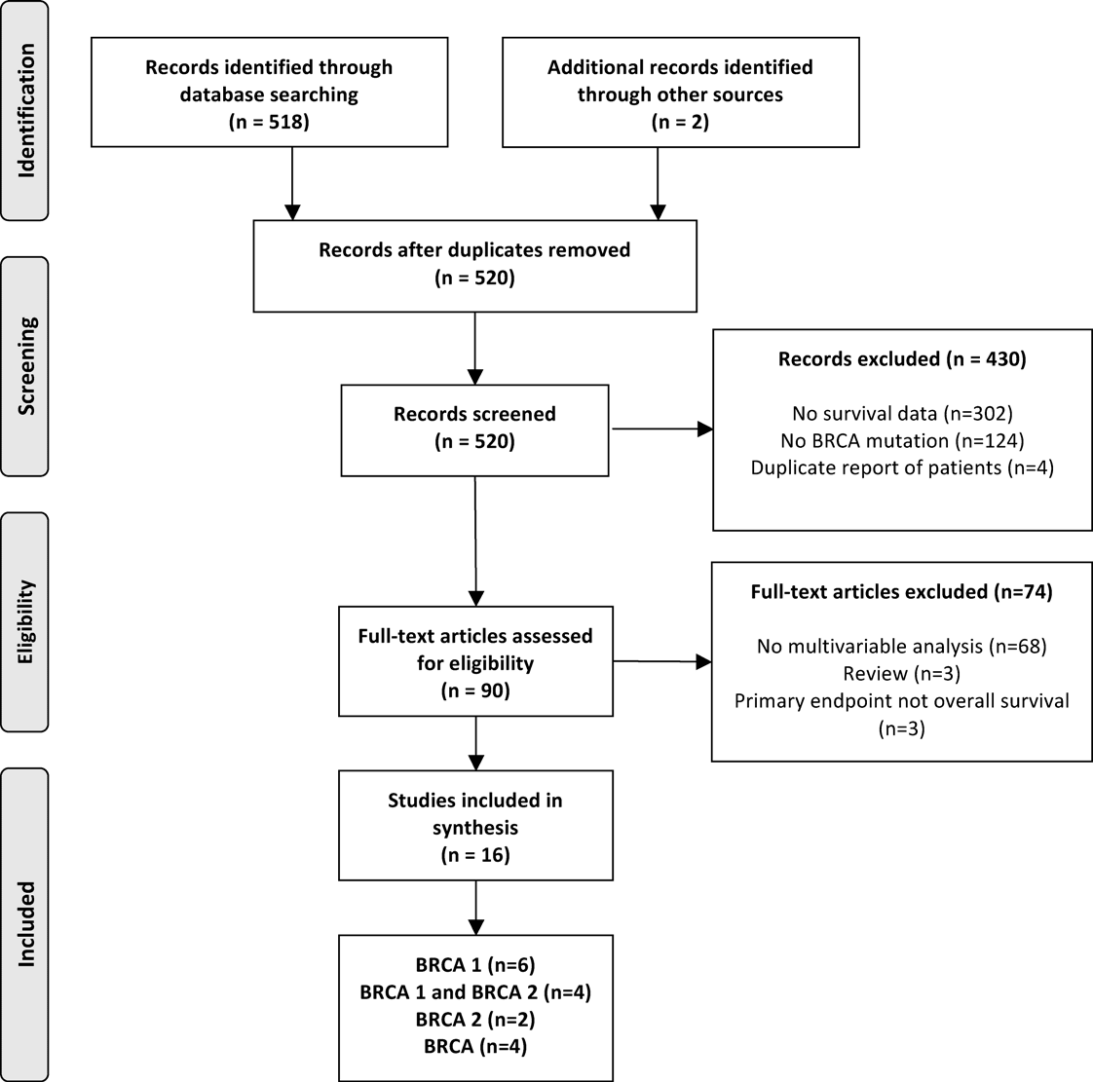
Selection of included studies.

A total of 16 studies were identified (Fig 1) and characteristics are presented in Table 1. These studies comprised a total of 10,180 patients (1,325 patients [13%] had *BRCA* mutations) with a mean or median follow-up of 69 months (range of medians/means 34 to 228 months). Six studies [6–11] reported outcomes for *BRCA1* alone, four [3, 12–14] for both *BRCA1* and *BRCA2*, two [15, 16] for *BRCA2* alone, and four studies [17–20] reported data for unspecified *BRCA* mutations.

**Table 1:** Characteristics of Included Studies.

Reference	Mutation	BRCA mut	BRCA-	Total patients	Study population	Age (mean or median)	Follow-up (mean or median)	Proportion ER+	Proportion PR+	Proportion metastatic	HR adjusted for
Bayraktar 2011	BRCA	114	113	227	High-risk women with TNBC referred for genetic testing	41	41	0%	0%	0%	Age, clinical stage, [*]
Bayraktar 2013	BRCA1/BRCA2	41	154	195	Women referred for BRCA testing	39	34	59%	43%	100%	Nodal status, Grade, bisphosphonates, tripple negativity
Brekelmans 2007	BRCA1/BRCA2	260	238	498	Cases and controls (comparable BC patients)	43	57	61%	59%	0%	T- and N-stage, grade, ER status, chemotherapy, endocrine therapy
Budroni 2009	BRCA2	44	464	508	Consecutive patients consenting for testing	50	nr	74%	56%	6%	Pathologic T stage, pathologic N stage, M, ER, PR
Cortesi 2010	BRCA1	80	931	1011	High and intermediate risk patients undergoing testing; sporadic BC as control	nr	72	67%	62%	0%	Stage, ER, PR, grade, age, chemotherapy
Goffin 2003	BRCA1	30	248	278	Ashkenazi Jewish women	53	96	63%	nr	41%	Tumor size, LN status, grade, p53 status
Gonzalez-Angulo 2011	BRCA	15	62	77	Patients with TNBC	51	43	0%	0%	0%	Pathological stage, grade, [*]
Goodwin 2012	BRCA1/BRCA2	166	1550	1716	Population-based cohort study	45	95	71%	70%	2%	Age, tumor and LN stage, grade, ER, PR status, year of diagnosis
Hamann 2000	BRCA1	36	49	85	Patients with hereditary BC	42	68	nr	nr	0%	Age, bilaterality
Huzarski 2013	BRCA1	233	3112	3345	Unselected women with newly diagnosed BC	42	89	59%	66%	49%	Year of birth, age, ER, PR, Her2, size, nodes, oophorectomy, tamoxifen, chemotherapy
Nilsson 2014	BRCA1/BRCA2	20	201	221	Unselected women offered BRCA1/2 germline testing	36	228	51%	58%	0%	Age, TNM stage, (neo)adjuvant chemotherapy, tumor grade, ER status
Rennert 2007	BRCA1/BRCA2	128	1189	1317	Incident cases of invasive breast cancer	56	nr	62%	nr	42%	Age, tumor size, LN status, metatases
Stoppa-Lyonnet 2000	BRCA1	42	150	192	Patients with BC and a family history of breast and/or ovarian cancer	42	58	56%	60%	26%	LN status
Verhoog 1998	BRCA1	49	196	245	BRCA1 carriers matched with controls with sporadic BC	40	nr	61%	61%	4%	Tumor stage, [**]
Verhoog 1999	BRCA2	28	112	140	BRCA2 carriers matched with controls with sporadic BC	46	nr	86%	81%	1%	Tumor stage, [**]
Veronesi 2005	BRCA	39	86	125	Patients with breast cancer and a family history of breast or ovarian cancer	40	69	68%	68%	43%	Age, grade

BC, breast cancer; ER, estrogen receptor; Her2, human epithelial growth factor receptor 2; LN, lymphnode; nr, not reported; OS, overall survival; PR, progesterone receptor.

* studies involved only patients with triple negative breast cancer;

** groups were matched according to age

### Influence of age at diagnosis and ER/PgR expression

**Fig 2:**
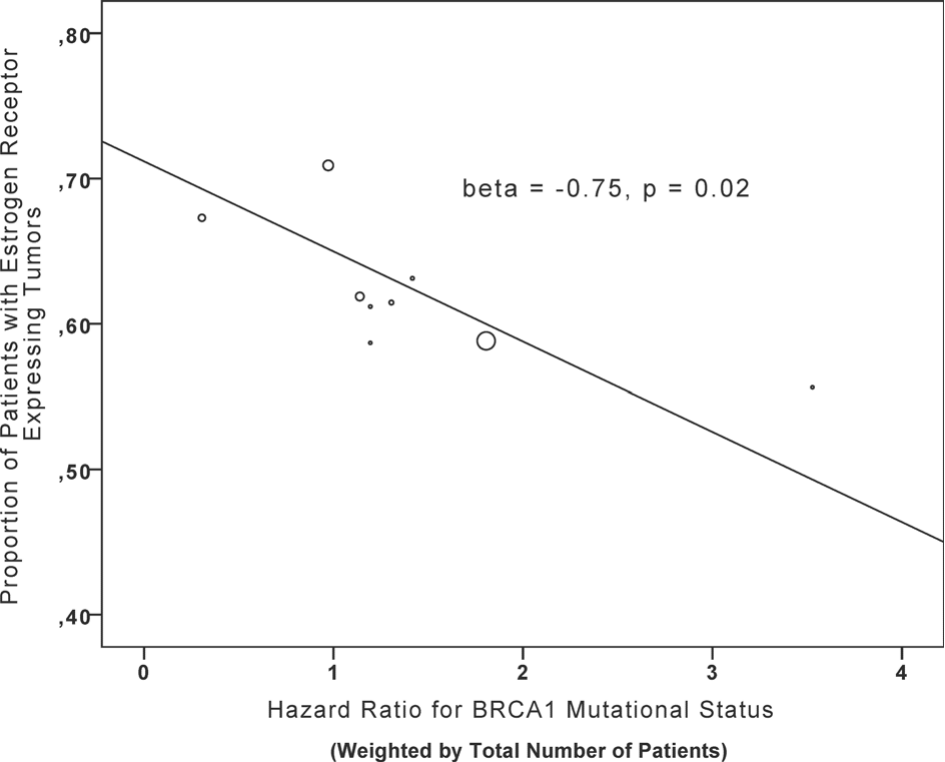
Meta-regression. Association of *BRCA1* germline mutational status and proportion of patients with estrogen receptor expressing tumors.

In meta-regression analysis, there was an inverse association between *BRCA1* mutation status and ER expression and overall survival (β = -0.75, p = 0.02, Table 2, Fig 2). No association was seen with age or PgR expression (Table 2). Also, no association was found for unspecified *BRCA* mutations or for *BRCA2* mutation status with age, ER, or PgR expression mutations (Table 2). Sensitivity analyses including only studies with HRs adjusted for age and hormone receptor expression yielded similar results (Table 3). Specifically, the magnitude of the association of ER and overall survival in BRCA1 mutation carriers remained similar (β = -0.80 and β = -0.79 for studies with HRs adjusted for hormonal receptors and age, respectively).

**Table 2:** Meta-regression (weighted by total sample size of studies).

	*BRCA*		*BRCA1*		*BRCA2*	
	β	*p*-value	β	*p*-value	β	*p*-value
Age	-0.39	0.15	-0.45	0.23	0.45	0.38
ER expression	-0.13	0.65	-0.75	0.02	-0.32	0.54
PgR expression	0.25	0.40	0.02	0.97	0.58	0.31

ER, estrogen receptor; PgR, progesterone receptor

**Table 3:** Meta-regression (weighted by total sample size of studies); studies with HRs with and without adjustment for age (upper part) and hormonal receptors excluded (lower part) only.

		BRCA		BRCA1		BRCA2	
		β	*p*-value	β	*p*-value	β	*p*-value
Studies with adjustment for age							
	Age	-0.45	0.22	-0.59	0.30	0.45	0.70
	ER expression	-0.15	0.70	-0.79	0.12	-0.87	0.33
	PgR expression	0.24	0.58	0.19	0.81	-1.00	nd
Studies without adjustment for age							
	Age	-0.31	0.54	-0.16	0.84	0.12	0.93
	ER expression	-0.06	0.91	-0.80	0.20	-0.08	0.95
	PgR expression	0.36	0.55	0.40	0.74	0.94	0.22
Studies with adjustment for hormonal receptors							
	Age	-0.62	0.10	-0.79	0.22	0.05	0.95
	ER expression	-0.07	0.86	-0.80	0.104	0.29	0.72
	PgR expression	0.36	0.34	0.13	0.84	0.91	0.092
Studies without adjustment for hormonal receptors							
	Age	-0.35	0.45	-0.56	0.32	1.00	nd
	ER expression	-0.56	0.24	-0.93	0.072	-1.00	nd
	PgR expression	-0.64	0.36	-1.00	nd	nd	nd

ER, estrogen receptor; PgR, progesterone receptor; nd, not determined

### Association of *BRCA*1/2 mutations with overall survival (OS)

Pooled analysis of all 16 studies reporting data for *BRCA1*, *BRCA2* or unspecified *BRCA* mutations showed no association between the presence of mutations and overall survival (HR 1.06, 95% CI 0.84–1.34, p = 0.61) (S1 Fig). There was evidence of inter-study heterogeneity (Cochran’s Q p = 0.001, I^2^ = 60%), which was mainly introduced by the study of Cortesi et al. [6]. Excluding this study did not significantly change the overall findings (HR = 1.16, p = 0.14), but reduced heterogeneity (Cochran’s Q p = 0.04, I^2^ = 43%).

When studying specific mutations, for the 10 studies evaluating *BRCA1*, no association with overall survival was observed (HR 1.21, 95% CI 0.91–1.61, p = 0.20, see S2B Fig). There was significant heterogeneity (Cochran’s Q p = 0.005, I^2^ = 62%) introduced by one outlier study, Cortesi et al. [6], which reported better outcomes for women with *BRCA1* mutations. After exclusion of this study *BRCA1* mutations were associated with a similar effect on overall survival (HR 1.34, 95% CI 1.12–1.60).

Similarly, no association with prognosis was observed for the studies evaluating *BRCA2* (HR 1.01, 95% CI 0.80–1.27, p = 0.95), without evidence of heterogeneity (Cochran’s Q p = 0.053, I^2^ = 0%, see S2B Fig).

No association between *BRCA* mutations and overall survival was found for the pooled analyses of four studies evaluating populations with unspecified *BRCA* mutations (HR 0.84, 95% CI 0.38–1.84, p = 0.66, see S2C Fig).

The overall results were unchanged when excluding studies for which reported HRs were not adjusted for age and hormonal receptors, respectively (S2 Table).

### Publication bias

Visual inspection of the Funnel plot did not indicate evidence of publication bias (S3 Fig).

## Discussion

The association between the presence of mutations in *BRCA1* and *BRCA2* genes and an increased risk of developing breast cancer is well known. Additionally, it has been established that after diagnosis of breast cancer, presence of *BRCA* mutation does not appear to influence cancer outcomes after adjustment for tumor stage. However it is less clear how age at diagnosis and ER or PgR expression contributes to the oncogenic phenotype of tumors wearing these mutations therefore affecting prognosis. In the present article we explored the hypothesis that *BRCA* mutation carriers with ER-negative tumors do better than women with *BRCA* wildtype and ER-negative tumors and found that ER-expression appears to be an effect modifier in patients with *BRCA1* mutations, but not among those with *BRCA2* or unspecified *BRCA* mutations. Furthermore, we confirm that *BRCA* mutation status does not affect survival.

These novel findings have relevant clinical implications. It may be reassuring for patients and their families that their long-term prognosis is not negatively influenced purely by the presence of a *BRCA* mutation. These data reinforce the concept that presence of germline mutations facilitate tumor initiation but do not influence tumor behavior. However, in the subgroup of women with *BRCA1* germline mutations and low or absent expression of hormone receptors prognosis may be less favorable. Yet, these data do not provide data to inform of treatment choice. In the recently reported Treating to New Targets (TNT) trial, patients with *BRCA* mutations and triple negative breast cancer had similar outcomes as those with sporadic triple negative breast cancer [21]. However, despite this finding, response to taxane and platinum-based chemotherapy was discordant in those with mutant and wildtype *BRCA* status. As such, the role of individualized therapy in *BRCA* mutation carriers remains an area where further research is warranted.

A mutation in a gene is relevant when it is necessary and sufficient to initiate and promote an oncogenic process. However, even in this situation, oncogenic mutations are not always linked with worse outcome [22]. During tumor evolution, acquired molecular alterations differentiate tumoral clones with a more aggressive phenotype. In this context, it is unclear which molecular alteration or combinations of molecular alterations facilitate this state; however, as our data show, germline mutations of these genes do not contribute to this phenotype. Our observations suggest that mutations in *BRCA2* but not *BRCA1* genes are linked with the initiation of the oncogenic process rather than with a clear role in the progression of the tumor or sensitivity to anti-cancer treatment.

An earlier meta-analysis of 11 studies comparing overall and disease-free survival rates between *BRCA1/2* mutation carriers and non-carriers found significantly lower short-term and long-term survival rates for *BRCA1* mutation carriers (HR = 1.92 and 1.33, respectively) while both short-term and long-term survival rates of *BRCA2* mutation carriers did not differ from non-carriers [23]. A more recent review of the literature and meta-analysis by Zhong and colleagues [24] identified 13 studies that examined the effects of *BRCA1/2* on breast cancer survival and found that *BRCA1* mutation carriers had worse overall survival than non-carriers (HR = 1.50, p = 0.009) whereas progression-free survival was not different. *BRCA2* mutation was not associated with breast cancer prognosis. Reasons for the difference in findings compared to our results may be due to the fact that both prior meta-analyses also included HRs from univariable analyses. It is known that breast cancer associated with *BRCA* mutations are more likely to be associated with young age and in the case of *BRCA1* mutations with triple negative phenotype. The unadjusted enrichment for breast cancer with these characteristics may lead to erroneous associations with worse outcomes among *BRCA* mutation carriers. In the work by Zhong et al. a subgroup analysis of studies using multivariable analyses, *BRCA1* mutation carriers only had borderline worse overall survival (HR = 1.40, p = 0.05) and also another recent review of the literature and meta-analysis did not find worse breast cancer survival in the adjuvant setting for BRCA1/2 mutation carriers [25].

Our study has limitations. Included studies used predominantly case-control methodology; thus, control for confounders is difficult with this design. To account for this problem, at least in part, we only included studies reporting HRs from multivariable analyses. However, the variables included in the various multivariable models were heterogeneous and adjustment is only able to control for measured confounders and not all studies reported HRs with adjustment for age at diagnosis or expression of hormonal receptors. To account for this we weighted all analyses by total sample size as studies reporting adjusted HRs for age and hormonal receptors comprised 83% and 77% of all patients. In addition, we performed sensitivity analyses excluding studies with HR not adjusted for the respective variables, what did not change the overall findings. However, the potential for residual confounding remains. Furthermore, our study is a meta-analysis of the literature rather of patient level data and there is a potential for selection bias by studies reporting positive results (although visual inspection of the Funnel plot did not indicate that this was a major issue). A further concern is the inter-study variability in a number of our analyses.

In conclusion, there is no apparent difference in overall survival in *BRCA* mutation carriers and non-carriers. However, there appears to be a strong and statistically significant association between ER expression and overall survival in patients with *BRCA1* germline mutations but not with age or PgR expression.

## Supporting Information

S1 Fig Overview of all studies (*BRCA1* and *BRCA2* data combined, if reported separately). (PDF)

S2 Fig Pooled estimates for *BRCA1* (A), *BRCA2* (B), and unspecified BRCA mutations (C).
(PDF)

S3 Fig Funnel plot. (PDF)

S1 Table PRISMA Checklist. (DOC)

S2 Table Sensitivity analyses of the association of overall survival and BRCA mutational status. (DOCX)
