# Supplementary figures and images for: Interaction between Hormonal Receptor Status, Age and Survival in Patients with BRCA1/2 Germline Mutations: A Systematic Review and Meta-Regression
Source: PLoS One. 2016 May 5;11(5):e0154789. doi: 10.1371/journal.pone.0154789 (PMC4858163; doi:10.1371/journal.pone.0154789)

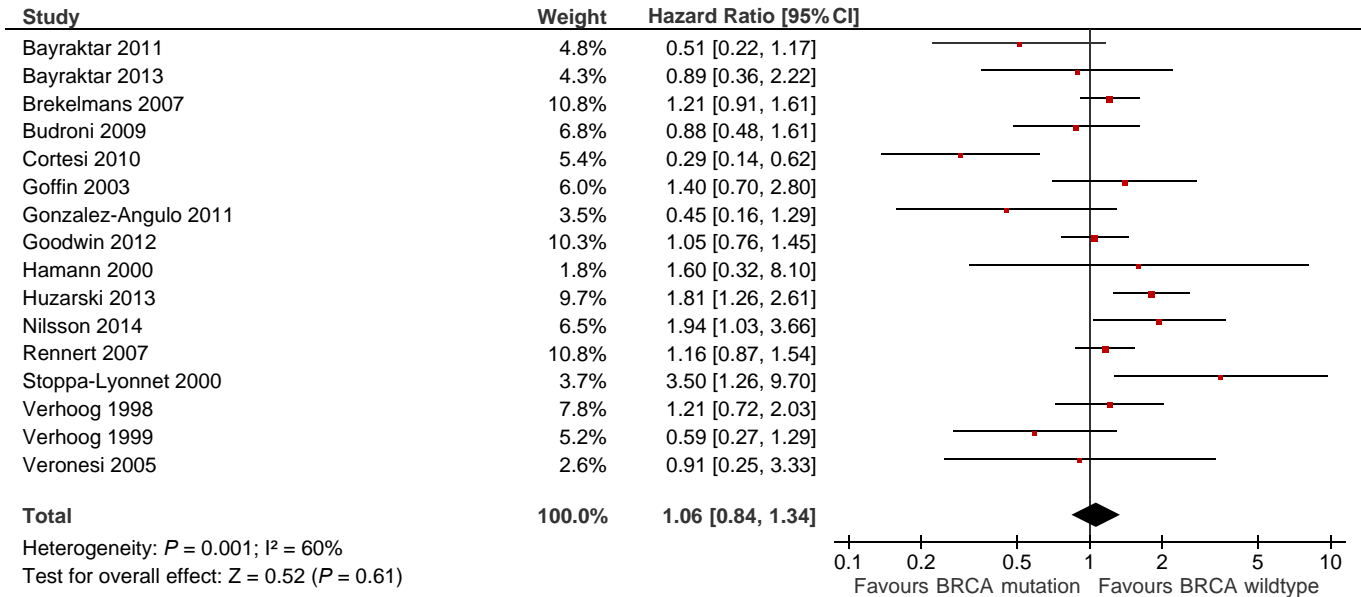

Supplement: S1 Fig — (PDF) [file pone.0154789.s001.pdf]

A

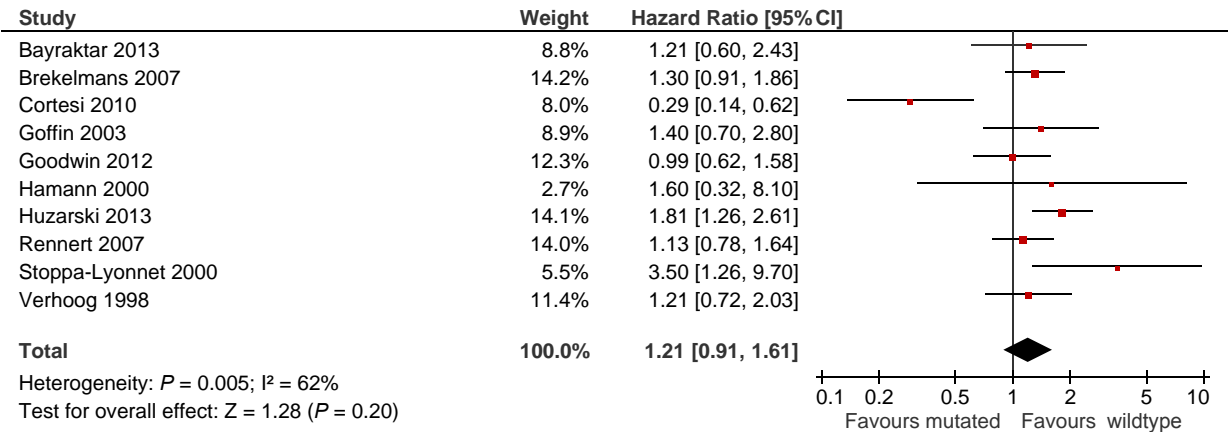

B

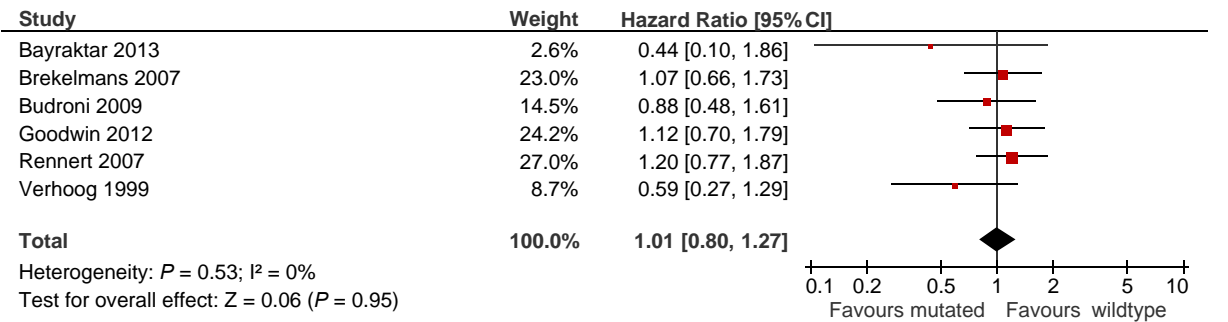

C

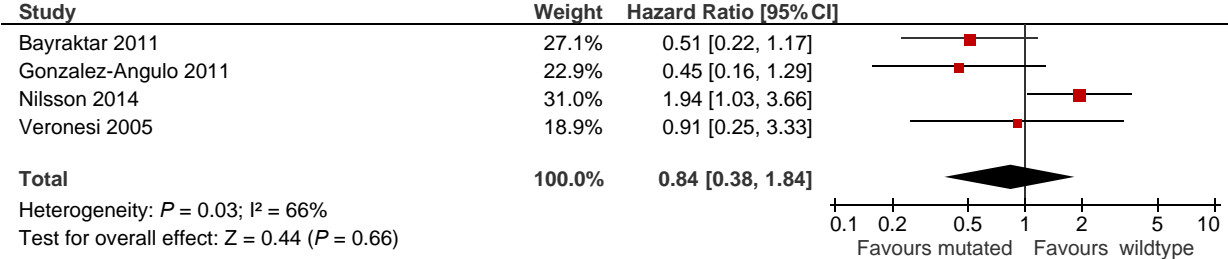

Supplement: S2 Fig — Pooled estimates for BRCA1 (A), BRCA2 (B), and unspecified BRCA mutations (C). (PDF) [file pone.0154789.s002.pdf]

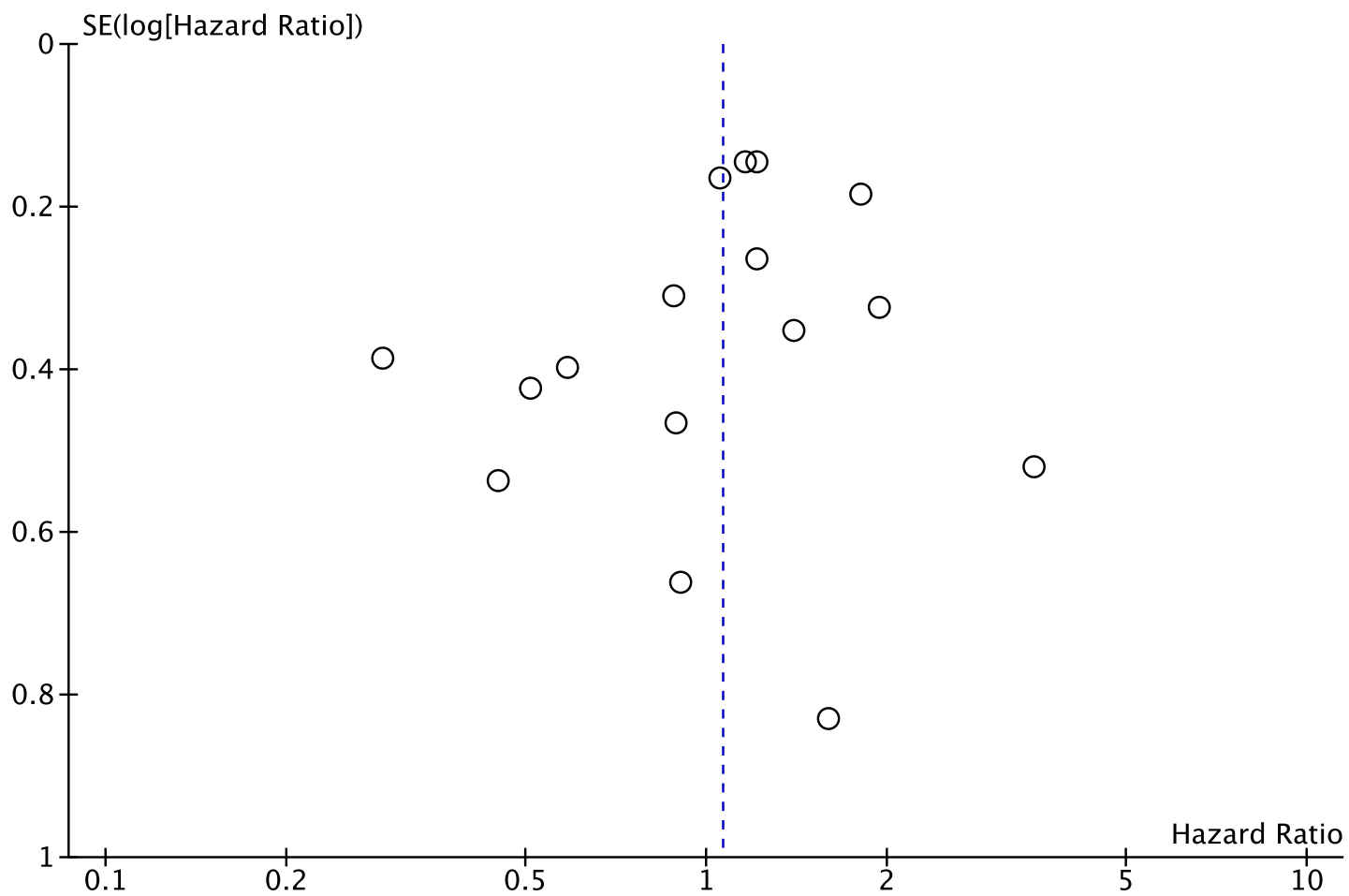

Supplement: S3 Fig — (PDF) [file pone.0154789.s003.pdf]
