# Supplementary material for: Interaction between Hormonal Receptor Status, Age and Survival in Patients with BRCA1/2 Germline Mutations: A Systematic Review and Meta-Regression
Source: PLoS One. 2016 May 5;11(5):e0154789. doi: 10.1371/journal.pone.0154789 (PMC4858163; doi:10.1371/journal.pone.0154789)
Supplement: S2 Table — (DOCX) [file pone.0154789.s005.docx]

|  | | | | |
| --- | --- | --- | --- | --- |
|  |  | **Number of studies** | **pooled HR (95% CI)** | ***p* for subgroup difference** |
| **BRCA (all studies)** | |  |  |  |
|  | HR adjusted for age | 10 | 1.01 (0.73 - 1.39) | 0.63 |
|  | HR not adjusted for age | 6 | 1.14 (0.78 - 1.65) |  |
| **BRCA1** | |  |  |  |
|  | HR adjusted for age | 6 | 1.04 (0.68 - 1.59) | 0.24 |
|  | HR not adjusted for age | 4 | 1.42 (0.91 - 1.96) |  |
| **BRCA2** | |  |  |  |
|  | HR adjusted for age | 3 | 1.03 (0.74 - 1.46) | 0.71 |
|  | HR not adjusted for age | 3 | 0.94 (0.65 - 1.36) |  |
| **BRCA unspecified** | |  |  |  |
|  | HR adjusted for age | 4 | 0.84 (0.38 - 1.84) | na |
|  | HR not adjusted for age | 0 |  |  |
| **BRCA (all studies)** | |  |  |  |
|  | HR adjusted for receptor status | 9 | 0.95 (0.67 - 1.33) | 0.30 |
|  | HR not adjusted for receptor status | 7 | 1.20 (0.90 - 1.61) |  |
| **BRCA1** | |  |  |  |
|  | HR adjusted for receptor status | 5 | 1.03 (0.64 - 1.66) | 0.40 |
|  | HR not adjusted for receptor status | 5 | 1.31 (0.99 - 1.75) |  |
| **BRCA2** | |  |  |  |
|  | HR adjusted for receptor status | 4 | 1.01 (0.75 - 1.34) | 0.79 |
|  | HR not adjusted for receptor status | 2 | 0.91 (0.46 - 1.79) |  |
| **BRCA unspecified** | |  |  |  |
|  | HR adjusted for receptor status | 3 | 0.81 (0.38 - 1.84) | 0.88 |
|  | HR not adjusted for receptor status | 1 | 0.91 (0.25 - 3.33) |  |
|  | |  |  |  |

**S2 Table.** Sensitivity analyses of the association of overall survival and BRCA mutational status

HR, hazard ratio; na, not applicable
